# Life-long tailoring of management for patients with hypertrophic cardiomyopathy: Awareness and decision-making in changing scenarios

**DOI:** 10.1007/s12471-016-0943-2

**Published:** 2016-12-22

**Authors:** M. Michels, I. Olivotto, F. W. Asselbergs, J. van der Velden

**Affiliations:** 1 Thoraxcenter, Department of Cardiology, Erasmus MC, Rotterdam, The Netherlands; 2 Careggi University Hospital, Florence, Italy; 3 University Medical Center Utrecht, Utrecht, The Netherlands; 4 VU University Medical Center, Amsterdam, The Netherlands; 5 Netherlands Heart Institute, Utrecht, The Netherlands

**Keywords:** Hypertrophic cardiomyopathy, Diagnosis, Imaging

## Abstract

Hypertrophic cardiomyopathy (HCM) is the most common genetic heart disease, characterised by complex pathophysiology and extensive genetic and clinical heterogeneity. In most patients, HCM is caused by mutations in cardiac sarcomere protein genes and inherited as an autosomal dominant trait. The clinical phenotype ranges from severe presentations at a young age to lack of left ventricular hypertrophy in genotype-positive individuals. No preventative treatment is available as the sequence and causality of the pathomechanisms that initiate and exacerbate HCM are unknown. Sudden cardiac death and end-stage heart failure are devastating expressions of this disease. Contemporary management including surgical myectomy and implantable cardiac defibrillators has shown significant impact on long-term prognosis. However, timely recognition of specific scenarios – including transition to the end-stage phase – may be challenging due to limited awareness of the progression patterns of HCM. This in turn may lead to missed therapeutic opportunities. To illustrate these difficulties, we describe two HCM patients who progressed from the typical hyperdynamic stage of asymmetric septal thickening to end-stage heart failure with severely reduced ejection fraction. We highlight the different stages of this complex inherited cardiomyopathy based on the clinical staging proposed by Olivotto and colleagues. In this way, we aim to provide a practical guide for clinicians and hope to increase awareness for this common form of cardiac disease.

## Introduction

Hypertrophic cardiomyopathy (HCM) is defined by increased left ventricular (LV) wall thickness that is not solely explained by abnormal loading conditions [1, 2]. It is a cause of sudden cardiac death (SCD) at young age and progression to end-stage heart failure (HF) is a feared complication [1]. Disease onset generally ranges between 20–50 years of age, thus affecting patients in the prime of their life. Until recently, HCM patients have been considered a sort of cardiological curiosity, ‘as rare as white ravens’. Although the first mutation was identified as early as 1989, thus paving the way for the molecular diagnosis of HCM, society may still be relatively unaware of the true burden of HCM [3]. Community-based echocardiographic studies have consistently reported a 1:500 prevalence of HCM worldwide, while Semsarian and colleagues recently argued that a genetic diagnosis might be even more common with an estimated prevalence of 1:200 [4]. This high prevalence may be partly explained by the identification of new sequence variants which may or may not be pathogenic. The advances and limitations of genetic screening in HCM were recently discussed by Ho and colleagues [3]. Though great care must be taken in the adjudication of pathogenicity for new variants, large-scale implementation of affordable genetic screening [3, 4] has led to increasing numbers of identified genotype-positive/phenotype-negative (G+/Ph−) individuals, i. e. of potential patients.

**Fig. 1:**
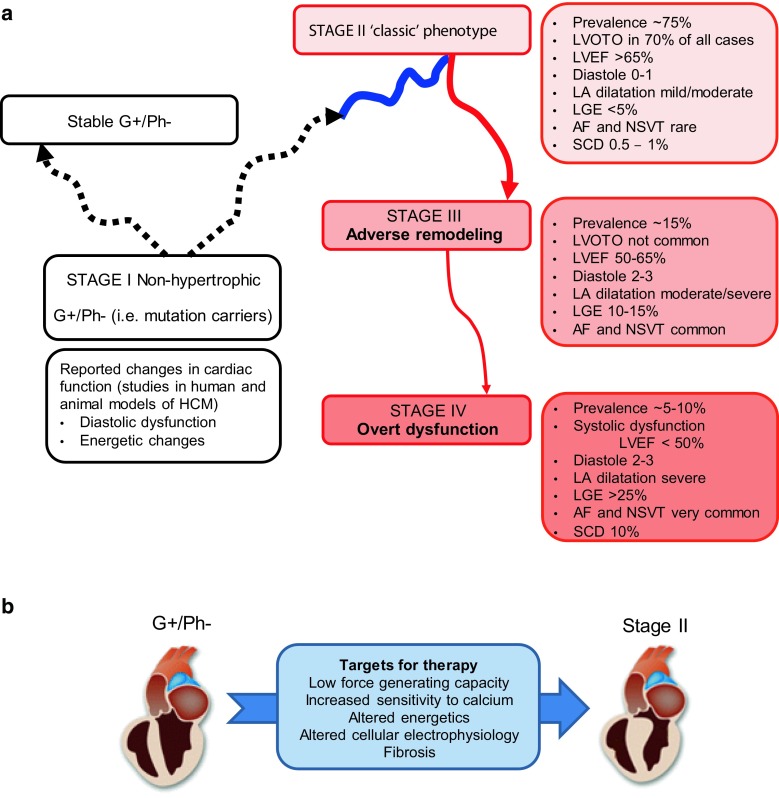
**a** Stages of hypertrophic cardiomyopathy (adapted from [4]) and clinical characteristics. Mutation carriers (genotype positive/phenotype negative, G+/Ph−) may develop HCM (i. e. move to stage II) or remain without cardiac symptoms throughout their life (i. e. stable G+/Ph−). **b** Studies in mouse models and human cardiac samples revealed cellular changes which may be target for therapy to prevent onset of HCM (*LVOTO* Left ventricular outflow obstruction, *LVEF* LV ejection fraction, *LA* Left atrial, *LGE* Late gadolinium enhancement, *AF* Atrial fibrillation, *NSVT* Non-sustained ventricular tachyarrhythmias, *SCD* Sudden cardiac death)

Advancing core clinical knowledge of HCM in the clinical community is important. The epidemiological relevance of HCM, its clinical heterogeneity and complex pathophysiology emphasise the need to regularly update clinicians on advances in basic and clinical research. Cardiologists are often capable of recognising and managing the classic manifestations of disease, but knowledge of the risk of disease progression and identification of the less typical (and less favourable) manifestations as HCM may be limited. As each clinical stage of HCM requires a different treatment strategy, recognising HCM at any given stage is critical for appropriate management and family screening. To accurately monitor disease progression and prescribe appropriate drug treatment, it is important to be aware of the changes that may occur during the different stages of disease development (Fig. 1a; [5]).

## Stages of HCM

As with any biological process or disease, HCM is a continuum. Any attempt to identify stages must thus be considered an oversimplification of countless exceptions and nuances present in the clinical spectrum. However, the following conceptual framework constitutes a clinically useful aid to understanding the long-term course of HCM and its complications, exemplified by the two cases presented below.

### Stage I – genotype-positive, phenotype-negative individuals

The status of G+/Ph− individuals, identified during family screening, is defined as Stage I (Fig. 1a). This group represents a major challenge, as based on past experience we now know that the same mutation will cause cardiomyopathy in one individual but may be harmless in a direct family member. Although LV hypertrophy – the hallmark of HCM – is absent, advanced echocardiographic characterisation, including tissue Doppler and strain imaging, reveals initial diastolic dysfunction and increased left atrial (LA) dimension in G+/Ph− individuals [6, 7]. Cardiac magnetic imaging (CMR) studies may show ancillary signs such as crypts, papillary muscle abnormalities and septo-apical bundles [8]. Current guidelines recommend long-term evaluation of G+/Ph− individuals with echocardiography and electrocardiography (ECG) [1], although, as indicated above, follow-up studies show development of LV hypertrophy only in a minority [9]. In the event of an abnormal ECG, it is advisable to perform CMR imaging, as apical or anterolateral hypertrophy may be missed on echocardiography and some individuals may present without hypertrophy but evidence of late gadolinium enhancement (LGE).

The recommended interval for follow-up clinical evaluations differs between guidelines; the American College of Cardiology Foundation/American Heart Association (ACCF/AHA) recommends screening every 12–18 months from age 12 to 18–21 years and at least every 5 years above the age of 21 [10]. The European Society of Cardiology (ESC) guidelines advise follow-up, but do not recommend a specific interval [1]. The proportion of G+/Ph− individuals that will develop overt disease is still uncertain. The likelihood of this increases with age, but conversion seems to be slow, both in children and adults [11, 12]. We currently see G+/Ph− individuals at 2‑year intervals when they have ECG and echocardiography. It is important to emphasise that the threshold to diagnose HCM in a first-degree relative of a proband or G+/Ph− individuals is less stringent than classic diagnostic criteria for novel patients, i. e. a maximal wall thickness (MWT) ≥ 13 mm is considered sufficient. SCD is extremely rare in the absence of LV hypertrophy, but has been described in isolated cases [13, 14]. In G+/Ph− individuals with a family history indicating a high SCD risk, periodic assessment of arrhythmias by means of exercise testing and Holter monitoring may be appropriate.

### Stage II – classic phenotype

Most HCM patients will present in stage II disease, the classic phenotype defined in the majority of patients by the presence of LV hypertrophy, normal or supernormal systolic function and LV outflow tract obstruction (LVOTO) at rest or during provocation (Fig. 1a). At this stage therapy is mainly determined by the presence and severity of symptoms associated with LVOTO or arrhythmias. For the management of symptoms and the assessment of SCD risk it is important to actively search for LVOTO in HCM patients without obstruction at baseline. Routine echocardiography should therefore include the Valsalva manoeuvre, and exercise echocardiography is recommended in symptomatic patients who are non-obstructive at rest [1].

Clinical and echocardiographic follow-up every 1–2 years is advised for asymptomatic HCM patients with maximum provoked peak LVOTO < 50 mm Hg, depending on the general context and associated risk factors. Current guidelines do not recommend aggressive treatment for patients with obstructive HCM who are asymptomatic. However, medical therapy is often prescribed for gradient control, particularly in active individuals. In symptomatic patients with LVOTO ≥ 50 mm Hg, the first step is medical therapy with non-vasodilating beta blockers. Verapamil can be used as an alternative for patients who are intolerant or have contraindications to beta blockers. Disopyramide is recommended in addition to beta blockers to improve symptoms. Invasive treatment (surgical myectomy or septal alcohol ablation) to reduce LVOTO is indicated in HCM patients who remain in NYHA Class III–IV with LVOTO ≥ 50 mm Hg despite optimal medical therapy. Surgeons and cardiologists who perform invasive treatment in HCM should work as part of a focused multidisciplinary team in order to choose the best treatment option. Symptomatic patients who are non-obstructive at rest or on provocation are treated with beta blockers, verapamil or diltiazem to control the heart rate. Judicious use of loop diuretics may be considered to reduce LV diastolic and pulmonary pressures.

In all HCM patients, the estimation of SCD risk is part of clinical management and should be re-evaluated at 1–2 year intervals or when there is a change in clinical status. Risk assessment should include clinical and family history, 48-hour ambulatory ECG, echocardiography and an exercise test. The exact value of CMR in the prediction of the SCD risk still needs to be determined. There is universal agreement on the indication for an implantable cardioverter defibrillator (ICD) for secondary prevention in cardiac arrest survivors. With regard to primary prevention, the approaches in Europe and the United States differ and the issue is currently a subject of debate. The recent ESC guidelines propose the use of a novel HCM SCD score, advising considering an ICD when the estimated 5‑year SCD risk exceeds 6% [1]. This score is currently being validated in independent cohorts, with somewhat conflicting results [15, 16]. The ACCF/AHA guidelines favour individual, non-parametric evaluation of major risk factors (family history of SCD, MWT ≥ 30 mm, syncope, presence of non-stained ventricular tachycardia on Holter monitoring and abnormal blood pressure response on exercise), and advise an ICD for primary prevention in the presence of ≥1 risk factor (family history of SCD, syncope or MWT ≥ 30 mm) or ≥2 risk factors [17].

Since HCM is an inherited disease, genetic testing is indicated to enable family screening by pre-symptomatic DNA testing. All HCM patients should be referred to a cardio-genetic centre, where cardiologists and clinical geneticists work in close collaboration and genetic testing is performed after comprehensive clinical phenotyping and counselling. Following the identification of a pathogenic mutation, genetic testing can be offered to identify family members at risk for disease. In HCM patients in whom DNA testing is inconclusive, first-degree family members should be offered cardiological screening by ECG and echocardiography. Before family members undergo clinical or genetic testing they should be counselled by a clinical geneticist to make sure they understand the possible consequences of testing.

### Stage III – adverse remodelling

In stage III, HCM is characterised by a gradual decrease of LV ejection fraction (LVEF), progression of diastolic dysfunction and LA dilatation, often with and the disappearance of LVOTO (Fig. 1a). A CMR-derived LVEF of 50–65% may already be associated with significant degrees of myocardial fibrosis, suggesting that progression to end-stage disease has begun. In the presence of congestive symptoms or increased filling pressures, standard HF therapy with diuretics, beta blockers, angiotensin converting enzyme (ACE) inhibitors, angiotensin receptor blockers (ARBs) and mineralocorticoid receptor antagonists (MRAs) may be considered. The presence of extensive LGE is important as it lowers the threshold for decision-making regarding ICD implantation [18]. At this stage, atrial fibrillation (AF) begins to emerge as the leading management challenge. AF is the most frequent arrhythmia in HCM, affecting more than 20% of patients. It not only worsens the symptoms of HF, but is also a marker of an unfavourable prognosis. All HCM patients with AF have an indication for oral anticoagulation, irrespective of the CHA2DS2-VASc score [19].

### Stage IV – overt dysfunction

Patients in whom the process of adverse remodelling proceeds further ultimately reach stage IV, in which the LVEF may drop below 50% and LV diastolic dysfunction is always severe, generally causing HF [19]. At this stage therapy with diuretics, beta blockers, ACE inhibitors, ARBs and MRAs in accordance with the ESC guidelines for the management of chronic HF is indicated. Progression to end-stage disease with systolic dysfunction is associated with a high risk of SCD and is an indication for ICD implantation [10, 19]. When HF becomes severe, referral to heart transplantation centres should be considered, irrespective of LVEF values, before refractory pulmonary hypertension occurs. Heart transplant in HCM candidates has an excellent prognosis. The use of LV-assist devices is possible but may be challenging due to small LV cavity dimensions, as HCM progression is more often hypokinetic-restrictive than hypokinetic-dilated.

## Genetic testing in HCM

Since the identification of the first HCM mutation in 1989, over 1500 pathogenic mutations in at least 11 genes encoding thick and thin myofilament protein components of the sarcomere have been identified [20]. A pathogenic mutation is found in about 50% of HCM patients [3]. In the Netherlands, three *MYBPC3* founder mutations (c.2373dupG, c.2827C > T, and c.2864_2865delCT) account for 35% of HCM cases [21]. Currently, the main clinical advantage of genotyping HCM patients is enabling family screening. The prognostic significance of genetic testing in individual HCM patients is less clear. Studies comparing genotype-positive and genotype-negative HCM patients have shown a favourable outcome for genotype-negative HCM patients [22–25]. The presence of double or compound sarcomere gene mutations in a patient is associated with earlier disease onset and more severe outcome [26].

## Lifestyle advice

As a general rule, conditions that reduce circulating blood volume should be avoided to prevent worsening of obstruction in the event of fever, diarrhoea and dehydration. Advice regarding an appropriate lifestyle may be extremely useful in reducing symptoms and risk in HCM patients, and may suffice in milder forms of the disease in which pharmacological therapy is not warranted. There is general consensus that patients should abstain from competitive sports, as well as from strenuous and protracted physical activities which can trigger arrhythmias and SCD (Class I, Class of recommendation in the 2014 ESC guidelines) [1]. The advisable level of exercise may be evaluated on an individual ba﻿sis by exercise echocardiography. Sporting activities should be pulsemeter-guided, with pre-specified maximum heart rates, allowing preference for endurance over stop-and-go disciplines or contact sports. Pregnancy is well tolerated in most HCM patients. However, adequate counselling should be provided and management should follow the 2011 ESC guidelines on the management of cardiovascular diseases during pregnancy. Similar considerations apply to non-cardiac surgery and invasive procedures in accordance with 2014 ESC/European Society Anaesthesiology (ESA) guidelines on non-cardiac surgery [27].

## Changing scenarios: a history of two patients

The difficulty of accurately managing the diverse paths that an HCM patient may travel is illustrated by the following case histories.

### Case 1

**Fig. 2:**
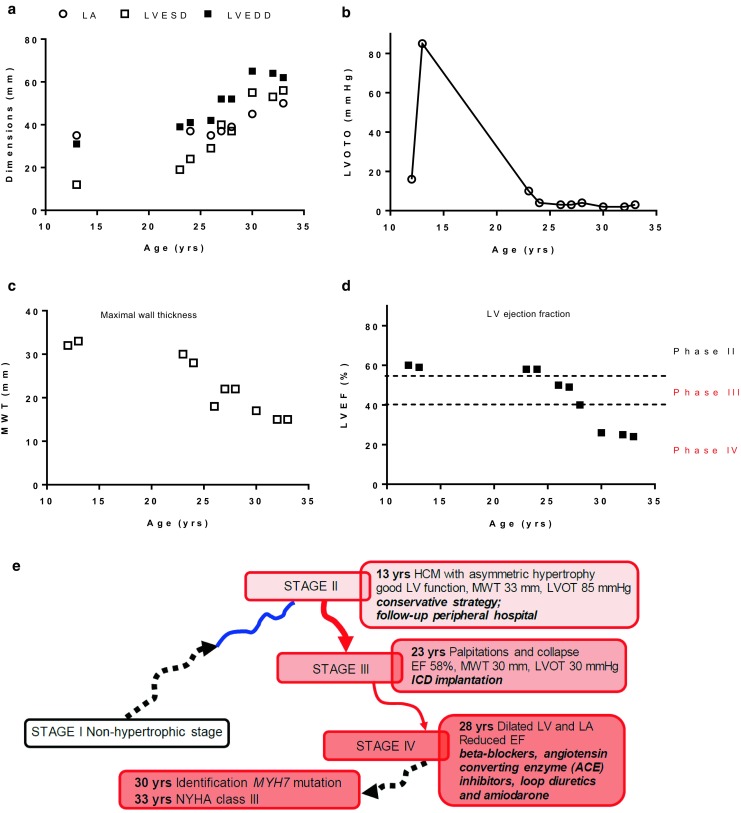
Progression of disease in patient case 1. **a** Progressive increase in cardiac dimensions, **b** a rise and subsequent decline in LVOTO, **c** a gradual decrease in MWT and **d** LV ejection fraction, **e** the different disease stages of the patient (*LA* Left atrium, *LVESD* Left ventricular end-systolic dimensions, *LVEDD* Left ventricular end-diastolic dimensions, *LVOTO* LV outflow tract obstruction, *MWT* Maximal wall thickness, *LVEF* LV ejection fraction [LVEF])

**Fig. 3:**
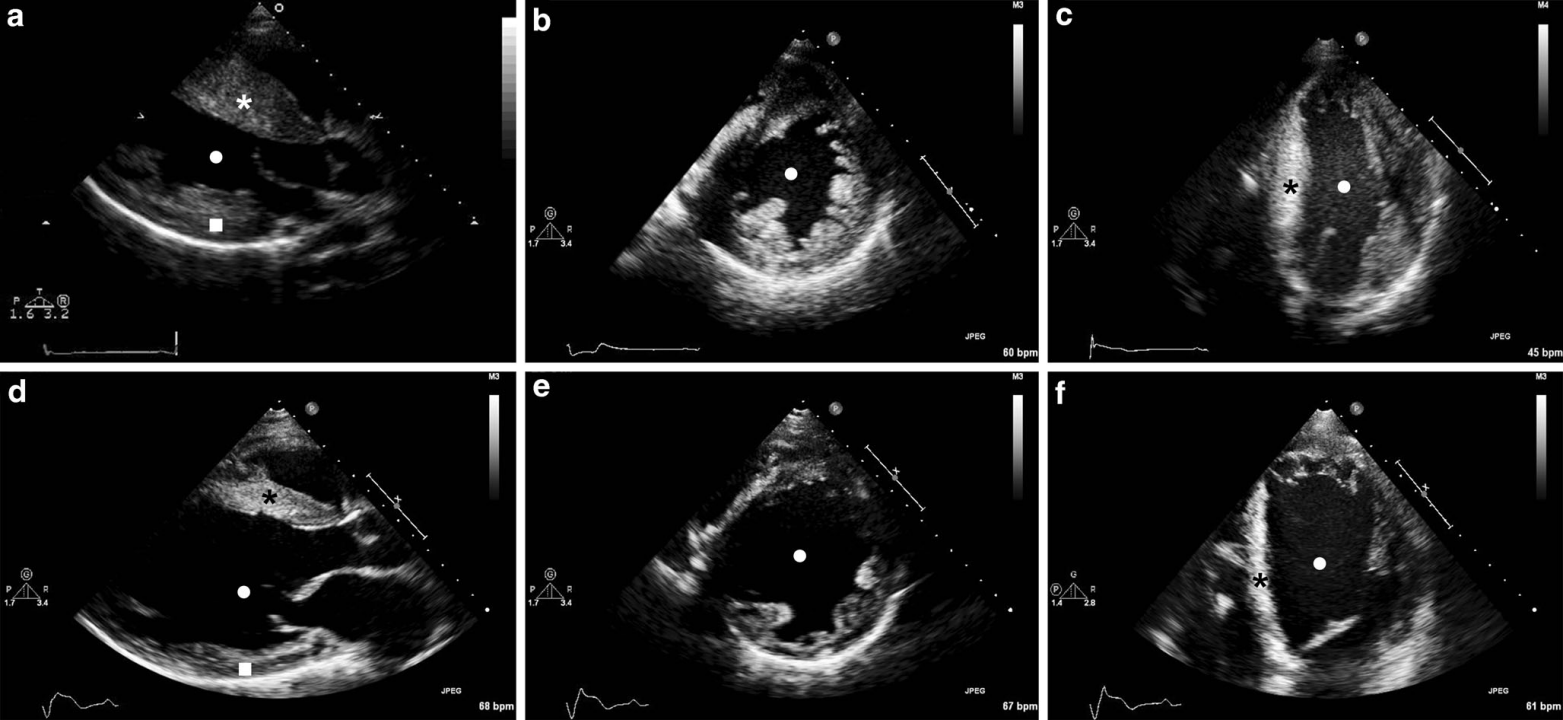
Echocardiographic follow-up of case 1. Transthoracic echocardiogram of case 1 in 2005 (**a**–**c**) and 2015 (**d**–**f**). During 10 years of follow-up there was a gradual decrease of septal (*) and left ventricular posterior wall (▪) thickness and an increase of left ventricular dimension (●)

The first patient is a 33-year-old man (Figs. 2 and 3). He was first seen at the age of 12 after he turned pale during exercise, but did not collapse. His family history was positive for HCM and SCD; his mother died at the age of 33. At that time, he was diagnosed with HCM following detection of asymmetrical LV hypertrophy and an MWT of 33 mm with normal systolic function; he had no LVOT gradient at rest or during the Valsalva manoeuvre. However, at the age of 13 a severe gradient of 85 mm Hg was noted on Valsalva (Fig. 2b). At this time he was clearly in stage II. Because of the absence of severe symptoms and his very young age, a conservative strategy was chosen and follow-up was performed at a peripheral hospital.

At the age of 23, he was referred to the HCM outpatient clinic of the Erasmus Medical Center with palpitations and syncope. At that time his echocardiogram showed an MWT of 30 mm, with normal systolic function and absence of LVOTO. Based on the presence of a positive family history for SCD, massive hypertrophy and syncope, he received an ICD for primary prevention of SCD. At the age of 28 he experienced his first appropriate ICD shock due to fast ventricular tachycardia (VT) at 214/min. At age 30 genetic testing revealed a pathogenic missense mutation in myosin heavy chain (*MYH7*;c.3133C > T;p.Arg1045Cys). At age 32 he experienced inappropriate ICD shocks caused by an isolation defect of the ICD lead, which was then replaced. At age 33 he experienced two further appropriate ICD shocks for fast VT.

Over 20 years of echocardiographic follow-up there was a gradual decrease in MWT, LVEF and LVOT gradient, and a gradual increase in LA diameter, LV end-diastolic dimension and LV end-systolic dimension (Figs. 2 and 3), suggesting progression from stage II to stage III. At age 33 the patient experienced his first episode of fluid retention leading to congestive HF. His LVEF is currently 25% and he is on a regimen of beta blockers, ACE inhibitors, loop diuretics, MRAs and amiodarone. He is in stage IV and NYHA class III, without signs of fluid retention, and has been referred to the cardiac transplantation programme. Thus far there have been no episodes of AF.

### Case 2

**Fig. 4:**
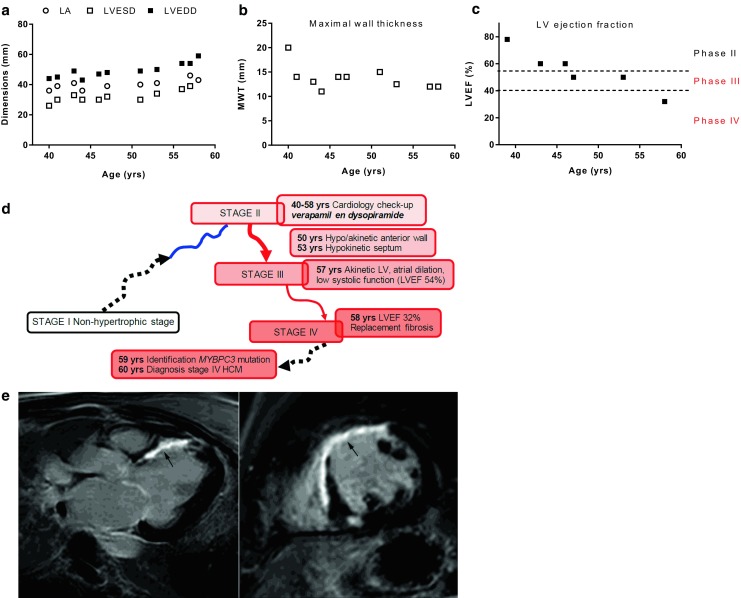
Progression of disease in patient case 1. **a** Progressive increase in cardiac dimensions (*LA* Left atrium, *LVESD and LVEDD* Left ventricular end-systolic and end-diastolic dimensions), **b** decline in maximal wall thickness (MWT) and **c** a decline in LV ejection fraction (LVEF). **d** The different disease stages of the patient. **e** Cardio magnetic imaging at stage IV of the disease showing a dilated heart and extensive late gadolinium enhancement (*arrow*)

The second patient is a 60-year-old woman, in whom mild asymmetrical hypertrophy was observed during a routine occupational check-up at age 39 (Fig. 4). Echocardiography showed a high LVEF (78%) and an MWT of 20 mm (stage II). She was followed-up yearly at a peripheral hospital. Echocardiography showed no LVOTO at rest, and provocation with Valsalva or exercise was never performed. Treatment with disopyramide and verapamil was started. From the age of 46 she had experienced recurrent chest pain. At age 50 the LV anterior wall was described as akinetic (stage II–III), and over subsequent years there was a gradual decrease of LVEF coupled with progressive dilatation of the LV and LA (stage III). At age 58 the patient was admitted to the emergency department with AF and a coronary angiogram was performed which excluded coronary artery disease as the cause of LVEF deterioration.

At age 58 the patient was seen at the outpatient clinic of the UMC Utrecht. CMR showed an LVEF of 32% and extensive LGE (Fig. 4e), suggesting replacement fibrosis and stage IV disease. Genetic testing identified a pathogenic splice-site mutation in the gene encoding myosin-binding protein C (*MYBPC3*; c.654 + 1G > A). This led to the conclusion that the patient had HCM, with LV dysfunction in the context of end-stage progression. The diagnosis was supported by residual mild, but significant LV hypertrophy (MWT 13 mm). The pattern of LGE together with a history of angina supported the presence of progressive ischaemic damage caused by microvascular dysfunction [28, 29] leading to replacement fibrosis.

These two cases illustrate how individual patients may progress through different stages of HCM during their lifetime, with profoundly different clinical profiles, risks and therapeutic needs. Unless all these stages are familiar to cardiologists, there is a substantial possibility that the disease will be misinterpreted and mismanaged over time.

## Optimal organisation for HCM patient care

Although HCM is the most common inherited cardiac disease, it is uncommon when compared with conditions such as coronary artery or valvular disease, and its devastating complications such as SCD and end-stage progression are rare. Fortunately, the majority of HCM patients have a favourable prognosis and a stable course. One of the ma﻿jor challenges in HCM care is the identification of patients at high risk for adverse outcome. During follow-up, special attention should be paid to so-called red flags of progression, reduction of LV function, LA dilatation, reduction of MWT and onset of AF. In addition, risk stratification for SCD should be performed periodically. HCM care needs close collaboration between cardiologists, clinical geneticists, thoracic surgeons, interventional cardiologists and electrophysiologists [30].

Decisions on when and how to intervene should be made at a centre with specific expertise in HCM care. A dedicated HCM heart team is critical in the interest of patients and their families. General cardiologists not specifically involved in HCM care should become familiar with the main issues such as identification of high-risk profiles and clinical screening of HCM families. However, they should closely liaise with experienced HCM centres whenever the diagnosis is uncertain, the symptoms are hard to explain and/or do not adequately respond to medical treatment, and when genetic counselling is required. Furthermore, peripheral centres should always seek advice from an HCM team when a major clinical decision (such as performing septal reduction therapy or implanting an ICD) is being considered. Finally, referral is mandatory for patients in Stage IV with severe HF symptoms, for timely consideration of heart transplantation. Because the end stage of HCM is characterised by a relatively preserved LVEF on comparison with classic models of HF, the severity of disease may be underestimated until transplant is no longer feasible due to refractory pulmonary hypertension. A particularly challenging group is the subset of patients identified in the paediatric age range, for whom specific HCM diagnostic criteria exist – but are not validated – and management options are poorly, if at all, supported by evidence [1]. Because of the complexity of genetic testing and the possible social and financial consequences, all patients and family members should be counselled before undergoing genetic testing. Genetic testing is done at cardio-genetic outpatient clinics, where clinical geneticists, genetic counsellors, social workers and cardiologists closely collaborate. Being part of an extended network revolving around HCM centres is the best guarantee for practising cardiologists and their patients, by promoting awareness and close interaction with multidisciplinary experts.

## Future perspectives in HCM research

The sequence and causality of the pathomechanisms that initiate and exacerbate HCM are unknown, and current treatment options are insufficient to prevent the disease. Longitudinal studies of *in vivo* cardiac function and *in vitro* studies (muscle preparations, HCM mouse models) are needed to elucidate the pathogenesis of HCM and identify novel drug targets (Fig. 1b). In the early stage of the disease (stage I), deficits are seen in energetic status of the heart, reflected by a reduced phosphocreatine/ATP ratio and reduced myocardial efficiency [31–33]. Moreover, Ho and colleagues [34] detected early pro-fibrotic signalling in patients with thick filament mutations before the onset of hypertrophy or detectable fibrosis on CMR. Thus, while mutation carriers (stage I) do not exhibit symptoms and do not show LV hypertrophy, the intrinsic properties of their heart muscle clearly differ from a healthy control population. Recent studies in human cardiac muscle revealed high sensitivity to calcium of the sarcomeres, which may underlie arrhythmias [35, 36], reduced contractile performance of single cardiomyocytes [37] and high energetic costs for muscle contraction [33]. These cellular pathomechanisms should be further explored [38, 39], and interventions aiming to correct these cellular deficits should be tested in an experimental setting (animal models). Such treatments include gene correction, and therapies targeting sarcomere function, ion channels or metabolism [40]. Subsequently, clinical trials are needed to build proof for novel preventive treatment strategies. Based on positive effects of the L‑type Ca^2+^ channel blocker diltiazem in mouse models of HCM, a clinical pilot study was performed in G+/Ph− individuals [41]. This double-blind, randomised, placebo-controlled clinical trial proved that preclinical treatment with diltiazem is safe and feasible and revealed that the drug treatment has a positive effect. Diltiazem prevented the progressive reduction in LV cavity size characteristic of HCM development. The improvement was lost within a year after treatment was interrupted. This study illustrates the strength of a well-designed clinical trial in G+/Ph− individuals. In order to further unravel the pathomechanisms of HCM, basic scientists and clinical cardiologists should work together with their ultimate goal being the development of a curative treatment of this common disease.
